# Definition of a Novel Pathway Centered on Lysophosphatidic Acid To Recruit Monocytes during the Resolution Phase of Tissue Inflammation

**DOI:** 10.4049/jimmunol.1500733

**Published:** 2015-06-22

**Authors:** Simon McArthur, Thomas Gobbetti, Dennis H. M. Kusters, Christopher P. Reutelingsperger, Roderick J. Flower, Mauro Perretti

**Affiliations:** * William Harvey Research Institute, Barts and London School of Medicine, Queen Mary University of London, London, EC1M 6BQ, United Kingdom;; † CARIM School for Cardiovascular Diseases, Maastricht University, 6200 MD Maastricht, the Netherlands; and; ‡ Department of Biochemistry, Maastricht University, 6200 MD Maastricht, the Netherlands

## Abstract

Blood-derived monocytes remove apoptotic cells and terminate inflammation in settings as diverse as atherosclerosis and Alzheimer’s disease. They express high levels of the proresolving receptor ALX/FPR2, which is activated by the protein annexin A1 (ANXA1), found in high abundance in inflammatory exudates. Using primary human blood monocytes from healthy donors, we identified ANXA1 as a potent CD14^+^CD16^−^ monocyte chemoattractant, acting via ALX/FPR2. Downstream signaling pathway analysis revealed the p38 MAPK-mediated activation of a calcium independent phospholipase A_2_ with resultant synthesis of lysophosphatidic acid (LPA) driving chemotaxis through LPA receptor 2 and actin cytoskeletal mobilization. In vivo experiments confirmed ANXA1 as an independent phospholipase A_2_–dependent monocyte recruiter; congruently, monocyte recruitment was significantly impaired during ongoing zymosan-induced inflammation in AnxA1^−/−^ or alx/fpr2/3^−/−^ mice. Using a dorsal air-pouch model, passive transfer of apoptotic neutrophils between AnxA1^−/−^ and wild-type mice identified effete neutrophils as the primary source of soluble ANXA1 in inflammatory resolution. Together, these data elucidate a novel proresolving network centered on ANXA1 and LPA generation and identify previously unappreciated determinants of ANXA1 and ALX/FPR2 signaling in monocytes.

## Introduction

Chronic inflammation is increasingly recognized as a major element of aetiopathogenesis, beyond more obviously inflammatory conditions such as rheumatoid arthritis and atherosclerosis, to include such socially and economically significant disorders as type 2 diabetes, Alzheimer’s disease, and cancer (1). Chronic inflammation can be characterized as a failure of resolution, the complex, active process of inflammatory reaction termination and restoration of homeostatic balance (2). Our understanding of the cellular and molecular interactions in resolution remains incomplete, but a key role is played by mononuclear phagocyte lineage cells (3).

One of the central functions of mononuclear phagocytes is the control and prevention of excessive neutrophil activation. Neutrophils, as the first cellular responders to infection or tissue damage, are critical for the body’s defenses, but it is essential that their actions are tempered: excessive or prolonged neutrophil action can be highly damaging to healthy bystander tissue, and, if not removed, effete neutrophils can themselves enter necrosis and further prolong inflammation (4). Thus, the phagocytic clearance of dead and dying neutrophils by monocytes and monocyte-derived macrophages is crucial for the progression to resolution.

Monocyte recruitment from the bloodstream to inflammatory sites is largely driven through migration toward chemoattractants (5). Many of these have now been identified, including complement factors and other classical chemoattractants, as well as chemokines such as CCL2 (6), and, importantly, proteins derived from neutrophils themselves (7). Indeed, patients with neutrophil granule content deficiencies show reduced monocyte recruitment during inflammation, despite normal responses to chemoattractants in vitro (8). One important neutrophil-derived protein is cathelicidin (LL-37 in humans, CRAMP in mice), recently shown to act through the G protein–coupled receptor ALX/FPR2 (9). ALX/FPR2 is a high-affinity receptor for the proresolving bioactive lipid mediator lipoxin A_4_ (LXA_4_) (10) and the low-affinity receptor for formylated peptides (11).

The role of ALX/FPR2 in monocyte recruitment is intriguing, as this receptor is highly promiscuous and capable of transducing signals for both proinflammatory ligands, such as LL-37, serum amyloid A, and β-amyloid (Aβ_1–42_), and for potent proresolution agents including LXA_4_ and the protein annexin A1 (ANXA1). ANXA1 in particular is not only highly abundant within neutrophils, comprising ∼2–4% of total cellular protein (12), but is a major proresolving agent, inducing both neutrophil apoptosis and their phagocytic clearance by macrophages (13, 14).

Given that ANXA1 shares with LXA_4_ a proresolving signature in human neutrophils and monocytes (15), and that LXA_4_ can promote nonphlogistic migration of monocytes (16), we employed an integrated in vitro and whole animal approach to investigate the actions of this protein upon monocyte recruitment and chemotaxis, defining a novel pathway operative during the second phase of inflammation, conceivably required for adequate resolution.

## Materials and Methods

### Animals

All procedures were performed under the United Kingdom Animals (Scientific Procedures) Act, 1986. Male C57BL/6 mice, male *alx/fpr2/3^GFP/GFP^* mice (hereafter referred to as alx/fpr2/3^−/−^) bearing a knocked-in GFP (17), and male *anxA1*^−/−^ mice (18) aged 6–8 wk were used for in vivo experiments. Both transgenic strains were fully backcrossed onto a C57BL/6 genetic background.

### Murine in vivo experiments

#### Zymosan-induced peritonitis.

Peritonitis was induced by i.p. injection of a suspension of 0.5 mg/100 μl zymosan particles (Sigma-Aldrich) in 0.9% NaCl and analyzed, as reported previously (19).

#### Polymicrobial sepsis.

Sepsis induced by cecal ligation and puncture was performed in 8-mo-old male C57BL/6 or alx/fpr2/3^−/−^ mice, utilizing our published protocol (19). Sham-operated mice underwent the same procedure, but without cecal ligation and puncture. Experiments were terminated 24 h postsurgery for ethical reasons.

#### Dorsal air pouch.

Subcutaneous dorsal air pouches were established and analyzed, as described previously (17).

### Cell culture

Primary human monocytes were extracted from whole blood of healthy donors using the RosetteSep negative selection assay (Stem Cell Technologies), according to the manufacturer’s protocols. Cells were resuspended in RPMI 1640 medium, supplemented with 0.1% BSA, 100 U/ml penicillin, and 100 μg/ml streptomycin for all assays. Extractions were performed immediately prior to use in experiments.

### Chemotaxis assays

#### Boyden chamber assay.

Monocyte chemotaxis was assessed in a 96-well plate format classical Boyden chamber assay (Neuroprobe, Gaithersburg, MD) consisting of a polycarbonate membrane containing 5-μm–diameter pores above a 30 μl chemoattractant chamber, with an incubation period of 90 min, as described previously (20). The human recombinant ANXA1 (hrANXA1) gradient established in this assay was confirmed as stable for at least 90 min in the absence of cells (Supplemental Fig. 1).

#### Three-dimensional chemotaxis assay.

Monocyte chemotaxis in three dimensions was assessed using an iBidi microslide^3D^ microfluidics system (iBidi, Munich, Germany), according to the manufacturer’s instructions. Briefly, monocytes were resuspended in 50% Matrigel (BD Biosciences) in RPMI 1640 supplemented with 1 mM CaCl_2_ and 0.5 mM MgCl_2_ in a central channel. Chemoattractant was added to media on one side of the channel only. Time-lapse video recording using a Nikon TE300 microscope (Nikon Instruments UK, Kingston-upon-Thames, U.K.) fitted with a Retiga X_i_ CCD camera (QImaging, Surrey, BC, Canada) under original magnification ×20 was used to track cell migration in a humidified chamber under 5% CO_2_, 95% air, images being captured every 20 s for 30 min prior to the addition of chemoattractant, followed by continued recording every 20 s for 30 min after the addition of hrANXA1 to one side of the channel. Migration was then quantified using the Chemotaxis 3D plugin to ImageJ 1.47 provided by iBidi, with movement of a minimum of 40 cells being assessed under each condition per donor.

### Flow cytometry

Isolated human monocytes were labeled with allophycocyanin-conjugated mouse monoclonal anti-CD14 and PE-conjugated mouse monoclonal anti-CD16 or isotype controls (all from eBioscience, Hatfield, U.K.), according to manufacturer’s protocols. Murine blood, peritoneal lavage, or air-pouch lavage cells were labeled with PE-conjugated rat monoclonal anti-Ly6G or anti-Gr1, PE-Cy5–conjugated rat monoclonal anti-CD115, and allophycocyanin-conjugated rat monoclonal anti-F4/80, or isotype controls (all from eBioscience), all according to manufacturer’s protocols. In all cases, 20,000 events were acquired using a FACSCalibur flow cytometer (BD Biosciences) and analyzed using FlowJo analysis software (Version 9.6.3; Tree Star, Stanford, CA). The human and murine gating strategies for cellular analysis are shown in Supplemental Fig. 2A and 2B, respectively. Actin polymerization was assessed through binding of AF488-labeled phalloidin (Invitrogen), according to manufacturer’s protocols.

### Immunofluorescence and confocal microscopy

Primary human monocytes were plated in six channel microslides (μ-Slide VI 0.4; iBidi), with hrANXA1 being added to one reservoir only. Cells were fixed with 2% formaldehyde in 0.1 M PBS prior to immunostaining with mouse monoclonal anti-FPR2 (Genovac, Freiburg, Germany) or rabbit polyclonal anti-iPLA_2_β (Cayman Chemical, Ann Arbor, MI), followed by secondary labeling with either AF488-conjugated goat anti-mouse IgG or AF594-conjugated goat anti-rabbit (both Invitrogen). Cells were then further labeled with 5 U/ml AF594- or AF488-conjugated phalloidin (Invitrogen) counterstained with DAPI and examined using a TCS SP5 confocal laser-scanning microscope (Leica Microsystems) fitted with 405, 488, and 594 nm lasers, and attached to a Leica DMI6000CS inverted microscope fitted with a 63× oil immersion objective lens (NA, 1.4 mm; working distance, 0.17 mm). Images were captured with Leica LAS AF 2.6.1 software and analyzed by using ImageJ software.

### Analysis of Akt, ERK1/2, and p38 MAPK activity

Monocytes, maintained in suspension, were incubated at room temperature for 1, 2, 3, 5, 10, or 15 min with 300 pM hrANXA1 before being transferred immediately onto ice. Expression of phospho- and total Akt, ERK1/2, and p38 MAPK (all Abs from Cell Signaling Technology, Danvers, MA) was assessed by Western blot, as described previously (15). Protein expression was detected by ECL using a FluorChem E imaging system (Proteinsimple, Santa Clara, CA), with quantification being performed using ImageJ.

### Phospholipase activity assays

Monocyte cytoplasmic phospholipase activity was assessed using a commercial enzyme activity kit (Cayman Chemical), according to the manufacturer’s protocol.

### ELISA

Commercial murine cytokine ELISAs were performed according to the manufacturer’s protocols (TNF-α, IL-1β, IL-10, MCP-1, and RANTES from eBioscience; KC from R&D Systems). Murine ANXA1 was measured using an in-house ELISA reported previously (15). Human monocyte lysophosphatidic acid (LPA) content was determined using a commercial assay kit (Echelon Biosciences, Salt Lake City, UT), according to the manufacturer’s instructions.

### RT-PCR and real-time RT-PCR

Total RNA was prepared from primary human monocytes using TRIzol reagent (Life Technologies, Paisley, U.K.) and then reverse transcribed with Superscript III reverse transcriptase (Life Technologies), according to the manufacturer’s protocols. Resultant cDNA was then analyzed by PCR using primer pairs specific for human LPA receptors 1–6 (21), alongside GAPDH as a positive control. Real-time PCR was performed in duplicate, using the Quantitect primer system (primer sets: LPA_1_ QT00021469, LPA_2_ QT01851318, LPA_4_ QT00235697, LPA_5_ QT00209503, and LPA_6_ QT01530648; all from Qiagen, Manchester, U.K.) and Power SYBR Green PCR Master Mix (Applied Biosystems, Warrington, U.K.). Reactions were performed in 384-well format using the ABI Prism 7900HT Sequence Detection System. The PCR conditions consisted of 95°C, 15 min [95°C 15 s − 55°C 30 s − 72°C 30 s] × 40, with a dissociation step [95°C 15 s/60°C 15 s/95°C 15 s] included after the PCR to confirm the absence of nonspecific products. Data were acquired and analyzed with SDS 2.3 (Applied Biosystems); fold change was calculated as 2^−ΔΔCt^.

### LPA_2_ small interfering RNA

Primary human monocytes were transfected with one of three different commercial small interfering RNA (siRNA) sequences designed to target LPA_2_ or an Allstars negative control siRNA sequence using Hiperfect transfection reagent (final concentration 2 nM; all from Qiagen, Hilden, Germany), alongside untransfected cells or cells treated with Hiperfect only. After 48 h, cellular chemotaxis to 300 pM hrANXA1 was assessed; a proportion of cells was analyzed for surface LPA_2_ receptor expression by immunofluorescence using a rabbit polyclonal anti-human LPA_2_ Ab (1:500 dilution; Santa Cruz Biotechnology, Dallas, TX) and an AF594-conjugated goat anti-rabbit secondary Ab (Invitrogen). Receptor expression on 20,000 events was analyzed using a FACSCalibur flow cytometer (BD Biosciences) and analyzed using FlowJo analysis software (Version 9.6.3; Tree Star). A similar proportion of cells collected after 48 h was analyzed for mRNA expression of LPA_1_, LPA_2_, LPA_4_, LPA_5_, and LPA_6_ by quantitative RT-PCR.

### Statistical analysis

All quantified in vitro data are derived from at least three independent donors, with experiments performed in triplicate, and are expressed as mean ± SEM. Murine in vivo experiments were performed with a group size of *n* = 4–6, sufficient to identify a 20% effect size with a power of 0.8, and are expressed as mean ± SEM. Data were tested for normality using the Shapiro–Wilk test and analyzed by one- or two-way ANOVA, as appropriate, with post hoc comparison using Tukey’s honest significant difference test. In all cases, a *p* value <0.05 was taken as indicating statistical significance.

## Results

### Annexin A1 attracts human monocytes via ALX/FPR2

**FIGURE 1.**
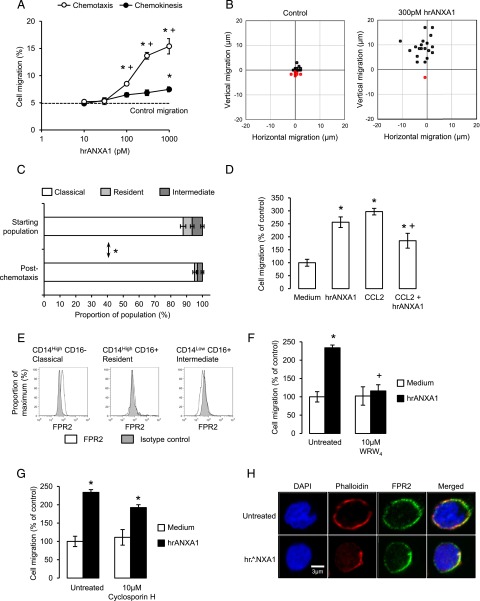
Annexin A1 is a potent chemoattractant of human classical monocytes in vitro, acting through ALX/FPR2. (**A**) Cumulative migration of human monocytes to hrANXA1 over a 90-min period, assessed using a 96-well Boyden chamber assay; data are mean ± SEM, and are representative of three independent donors. *p < 0.05 versus control migration, ^+^*p* < 0.05 versus chemokinesis. (**B**) End-point migration of human monocytes embedded in 50% Matrigel after 30-min exposure to medium or an increasing gradient of hrANXA1 (maximum 300 pM). Black points represent net positive migration; red points represent net negative migration. Data are representative of three independent donors. (**C**) Human monocyte population subtypes prior to and after migration toward 300 pM hrANXA1; data are mean ± SEM of three independent donors. *p < 0.05 between classical monocyte fractions. (**D**) Chemotaxis of human monocytes toward 300 pM hrANXA1, 200 pM hrCCL2, or a mixture of 300 pM hrANXA1 and 200 pM hrCCL2; data are mean ± SEM of three independent donors. *p < 0.05 versus medium control, ^+^*p* < 0.05 versus migration to hrCCL2 alone. (**E**) Surface expression of ALX/FPR2 by different human monocyte subtypes, defined by relative expression of CD14 and CD16; shaded histogram is IgG1 isotype control, clear histogram is FPR2, data are representative of three independent donors. (**F**) Chemotaxis of human monocytes toward 300 pM hrANXA1 with or without 10-min preincubation with the selective ALX/FPR2 antagonist WRW_4_ at 10 μM; data are mean ± SEM of three independent donors. *p < 0.05 versus medium control, ^+^*p* < 0.05 versus hrANXA1 treatment alone. (**G**) Chemotaxis of human monocytes toward 300 pM hrANXA1 with or without 10-min preincubation with the selective FPR1 antagonist cyclosporin H at 10 μM; data are mean ± SEM of three independent donors. *p < 0.05 versus medium control. (**H**) Confocal microscopic analysis of ALX/FPR2 localization in human blood monocytes after exposure to a hrANXA1 gradient (maximum concentration 300 pM). Images are representative of cells from three independent donors. Scale bar, 3 μm.

Recruitment of monocytes from the circulation is critical for resolution and termination of acute inflammation (2). The proresolving protein ANXA1 is a significant component of inflammatory exudates (22); hence, we investigated its monocyte chemoattractive potential. Using the classical Boyden chamber assay, we demonstrated a clear concentration-dependent chemotactic action of hrANXA1 upon human peripheral blood monocytes, effects being apparent at concentrations from 100 pM (Fig. 1A, Table I). This chemotactic effect was confirmed through use of a three-dimensional chemotaxis test, in which cells suspended in a 50% Matrigel matrix were exposed to unidirectional 300 pM hrANXA1 for 30 min. Untreated monocytes showed a low degree of random movement (Rayleigh test for uniformity of direction, *p* = 0.08), whereas cells exposed to hrANXA1 showed a striking net movement toward the protein (Rayleigh test, *p* = 2.12 × 10^−7^; Fig. 1B, Table II, Supplemental Videos 1, 2). In comparison, chemotaxis of human neutrophils toward hrANXA1 was negligible (Supplemental Table I).

**Table I.** Checkerboard analysis of monocyte chemotaxis toward hrANXA1

hrANXA1 in Lower Well (pM)	hrANXA1 in Upper Well (pM)				
	0	30	100	300	1000
0	5.1 ± 0.3	5.5 ± 0.1	6.5 ± 0.4	6.9 ± 0.5	7.5 ± 0.5*
30	5.3 ± 0.3	5.7 ± 0.1	5.4 ± 0.1	5.2 ± 1.4	6.6 ± 0.5
100	8.5 ± 0.6*	6.7 ± 0.5	6.5 ± 0.7	6.4 ± 0.8	6.1 ± 1.9
300	13.6 ± 1.4*	7.7 ± 0.4*	7.5 ± 0.3*	6.3 ± 0.4	6.2 ± 0.5
1000	15.4 ± 0.8*	8.9 ± 0.3*	9.6 ± 0.8*	10.8 ± 0.9*	6.9 ± 0.7

Data are mean ± SEM of three independent donors.

* *p* < 0.05 versus control migration.

**Table II.** Characteristics of migrating monocytes in three-dimensional migration assay

	Control	hrANXA1 (300 pM)
Velocity (μm/min)	0.90 ± 0.12	3.96 ± 0.27*
Accumulated distance (μm)	28.70 ± 2.90	119.19 ± 7.59*
Euclidean distance (μm)	1.61 ± 0.17	9.57 ± 0.93*

Data are mean ± SEM of three independent donors.

* *p* < 0.05 versus unstimulated control cells.

Three principal classes of circulating monocytes have been described in healthy humans (23), as follows: CD14^high^CD16^−ve^ (classical cells), CD14^high^CD16^+ve^ (resident cells), and CD14^low^CD16^+ve^ (intermediate cells). Among these, CD14^high^CD16^−ve^ monocytes are the largest population and fulfill the principal resolving roles of monocytes; hence, we hypothesized these would be predominantly recruited by hrANXA1. Comparison of subtype distribution (gating strategy shown in Supplemental Fig. 2A) within cells of the same donor prior to and after chemotaxis toward hrANXA1 revealed a significant and selective increase in CD14^high^CD16^−ve^ cell proportion, with an accompanying decrease in the other subtypes (Fig. 1C), confirming classical monocytes as the principal responders to ANXA1. To assess ANXA1 chemoattractant potency, we compared monocyte migration toward 300 pM hrANXA1 with that toward CCL2 (200 pM). Migration toward either stimulus alone was of comparable magnitude, but, intriguingly, migration toward both together was significantly lower than to either alone (Fig. 1D).

As many of the bioactions of ANXA1 are mediated through ALX/FPR2 (24), we examined expression of this receptor on different monocyte subsets, confirming significant expression solely on CD14^high^CD16^−ve^ cells (Fig. 1E), as previously reported (25). The importance of ALX/FPR2 was further confirmed in experiments in which pretreatment of monocytes with the selective antagonist WRW_4_ abrogated hrANXA1-induced chemotaxis (Fig. 1F), an effect not seen with an antagonist specific for the closely related FPR1, cyclosporin H (Fig. 1G). Supporting these data, confocal microscopy examination of monocytes exposed to unidirectional 300 pM hrANXA1 revealed a clear concentration of ALX/FPR2 expression at the leading edge of migrating cells (Fig. 1H).

### Binding of ANXA1 to ALX/FPR2 triggers activation of phospholipase metabolism

**FIGURE 2.**
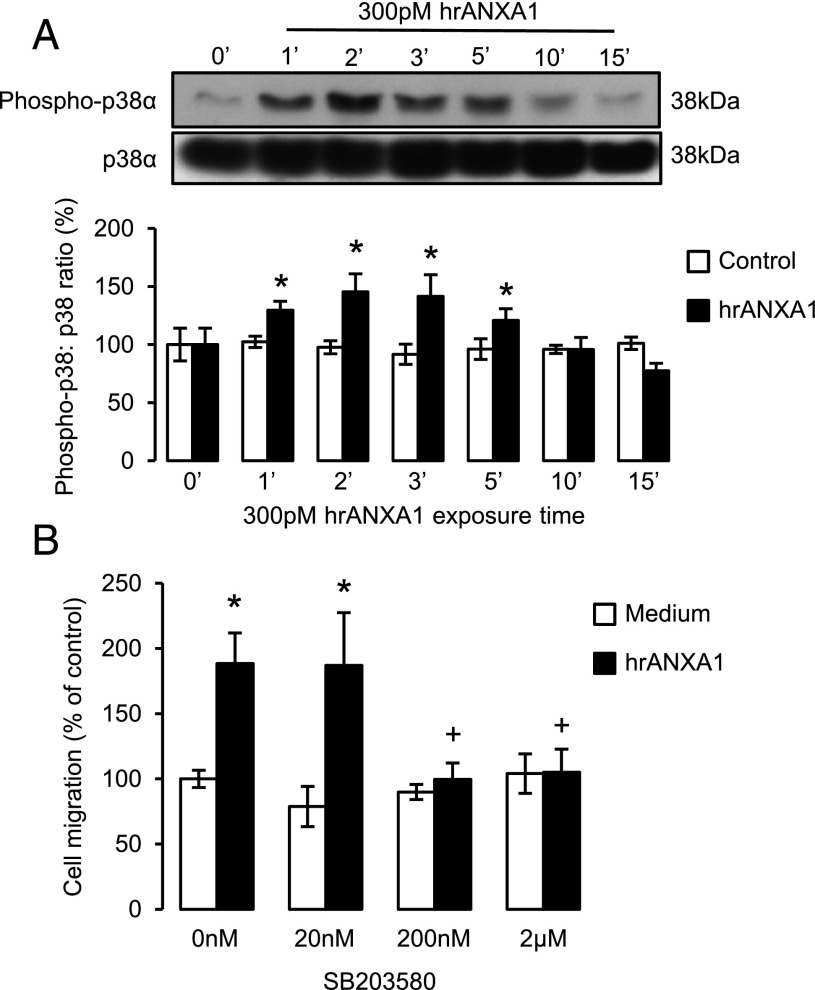
Chemotaxis of monocytes toward hrANXA1 requires sequential activation of p38 MAPK and calcium iPLA_2_. (**A**) Representative Western blot analysis of phospho-p38α and total p38α following exposure of human monocytes for 0, 1, 2, 3, 5, 10, and 15 min to 300 pM hrANXA1. Densitometric analysis data are mean ± SEM of three independent donors. *p < 0.05 versus respective 0-min control. (**B**) Chemotaxis of human monocytes toward 300 pM hrANXA1 with or without 10-min preincubation with the p38 MAPK inhibitor SB203580; data are mean ± SEM of three independent donors. *p < 0.05 versus medium control, ^+^*p* < 0.05 versus hrANXA1 treatment alone.

Several distinct signaling systems are known to be involved in the regulation of monocyte chemotaxis, including the PI3K/Akt, p38, and ERK1/2 pathways; accordingly, we investigated the engagement of these pathways in our defined experimental settings. Exposure of primary human monocytes to 300 pM hrANXA1 induced a rapid and short-lived increase in phosphorylated p38 MAPK, optimal between 1 and 5 min posttreatment (Fig. 2A), whereas, in contrast, no response was observed for Akt or ERK1/2 (data not shown). Functionally, pretreatment of monocytes for 10 min with the p38 MAPK inhibitor SB203580—at concentrations specific for p38α, the predominant monocyte/macrophage isotype (26)—blocked hrANXA1-driven chemotaxis (Fig. 2B).

**FIGURE 3.**
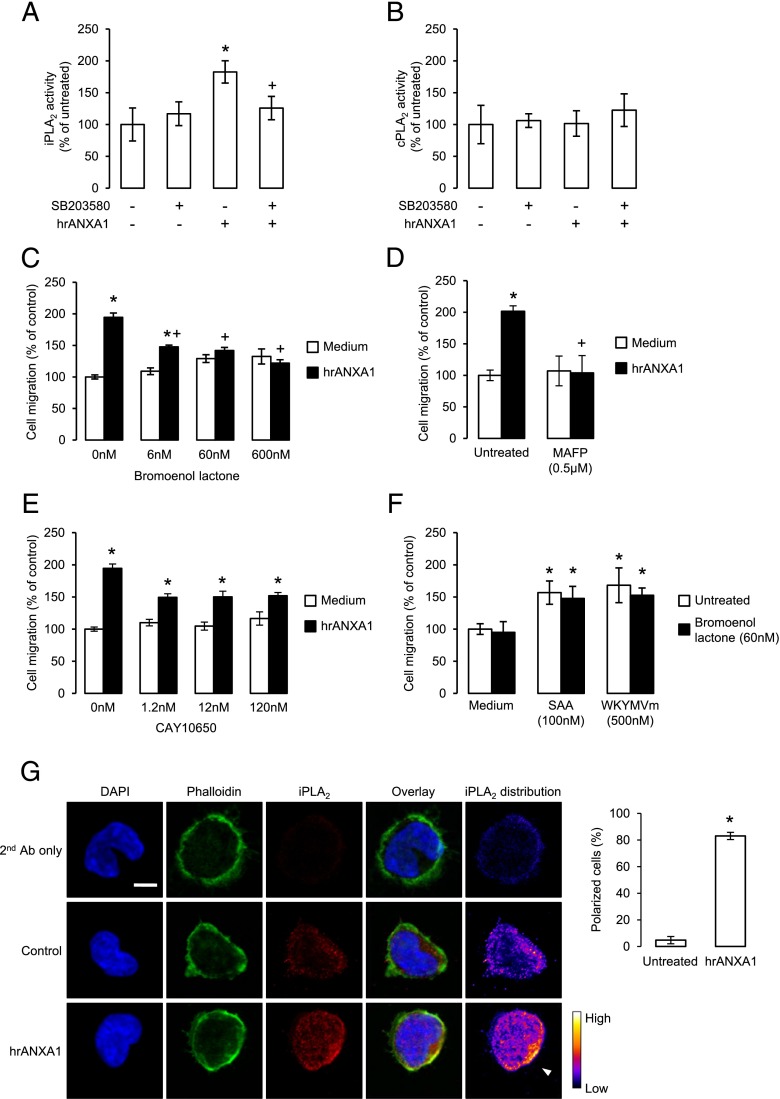
Chemotaxis of monocytes toward hrANXA1 requires activation of calcium iPLA_2_, but not cPLA_2_. (**A**) Human monocyte iPLA_2_ activity 30 min poststimulation with 300 pM hrANXA1, with or without 10-min pretreatment with the p38 MAPK inhibitor SB203580; data are mean ± SEM of four independent donors. *p < 0.05 versus untreated control, ^+^*p* < 0.05 versus hrANXA1 treatment. (**B**) Human monocyte cPLA_2_ activity 30 min poststimulation with 300 pM hrANXA1, with or without 10-min pretreatment with the p38 MAPK inhibitor SB203580; data are mean ± SEM of four independent donors. (**C**) Chemotaxis of human monocytes toward 300 pM hrANXA1 with or without 10-min preincubation with the iPLA_2_ inhibitor bromoenol lactone; data are mean ± SEM of three independent donors. *p < 0.05 versus medium control, ^+^*p* < 0.05 versus hrANXA1 treatment alone. (**D**) Chemotaxis of human monocytes toward 300 pM hrANXA1 with or without 10-min preincubation with the iPLA_2_ inhibitor methyl arachidonyl fluorophosphonate (MAFP); data are mean ± SEM of three independent donors. *p < 0.05 versus medium control, ^+^*p* < 0.05 versus hrANXA1 treatment alone. (**E**) Chemotaxis of human monocytes toward 300 pM hrANXA1 with or without 10-min preincubation with the cPLA_2_ inhibitor CAY10650; data are mean ± SEM of three independent donors. *p < 0.05 versus medium control. (**F**) Chemotaxis of human monocytes toward standard culture medium, 100 nM serum amyloid A or 500 nM WKYMVm with or without 10-min preincubation with the iPLA_2_ inhibitor bromoenol lactone (60 nM); data are mean ± SEM of three independent donors. *p < 0.05 versus medium control. (**G**) Confocal microscopic analysis of iPLA_2_ localization in human monocytes exposed to a hrANXA1 gradient (maximum concentration 300 pM), showing DAPI nuclear counterstain (blue), phalloidin-identified filamentous β-actin (green), and iPLA_2_ (red); false color images represent the distribution of iPLA_2_ immunostaining throughout the cell. Arrowhead indicates point of polarization; images are representative of three independent donors. Scale bar, 5 μm. Graph represents the proportion of monocytes exhibiting polarized iPLA2 distribution upon exposure to hrANXA1; data are mean ± SEM of three independent donors. *p < 0.05 versus untreated control cells.

There is literature associating ANXA1 biology with the enzyme cytosolic phospholipase A_2_ (cPLA_2_), a molecule that alongside the related calcium-independent phospholipase A_2_ (iPLA_2_) is known to be important in monocyte chemotaxis to CCL2 (27). Consequently, we investigated the potential importance of these two enzymes in cell chemotaxis evoked by application of low-dose ANXA1. Stimulation of human monocytes for 15 min with hrANXA1 (300 pM) induced a marked upregulation in iPLA_2_, but not cPLA_2_, activity, an effect sensitive to pretreatment with the p38 inhibitor SB203580 (Fig. 3A, 3B), indicating that iPLA_2_ lies downstream of p38. Activation of iPLA_2_ was directly related to chemotaxis, as incubation of monocytes with either of the selective iPLA_2_ inhibitors bromoenol lactone or methylarachidonyl fluorophosphonate prevented their migration toward hrANXA1 in a concentration-dependent manner (Fig. 3C, 3D). In contrast, application of the specific cPLA_2_ inhibitor CAY10650 had no effect on monocyte chemotaxis toward hrANXA1 (Fig. 3E). Importantly, application of bromoenol lactone was unable to modify the chemotactic response of human monocytes to the ALX/FPR2 ligands serum amyloid A or WKYMVm, indicating that activation of iPLA_2_ might be selective for ANXA1-triggered ALX/FPR2 signaling (Fig. 3F). Confocal microscopic analysis of human monocytes revealed that exposure to unidirectional hrANXA1 resulted in accumulation of iPLA_2_β immunoreactivity to the leading edge of the monocyte (Fig. 3G), an effect occurring together with cell polarization (Fig. 3G, bar graph).

Collectively, these experiments identify ANXA1 and ALX/FPR2 as a novel chemotactic determinant pair for monocyte chemotaxis and reveal the selective involvement of iPLA_2_.

### The iPLA_2_ product LPA underlies chemotactic migration

**FIGURE 4.**
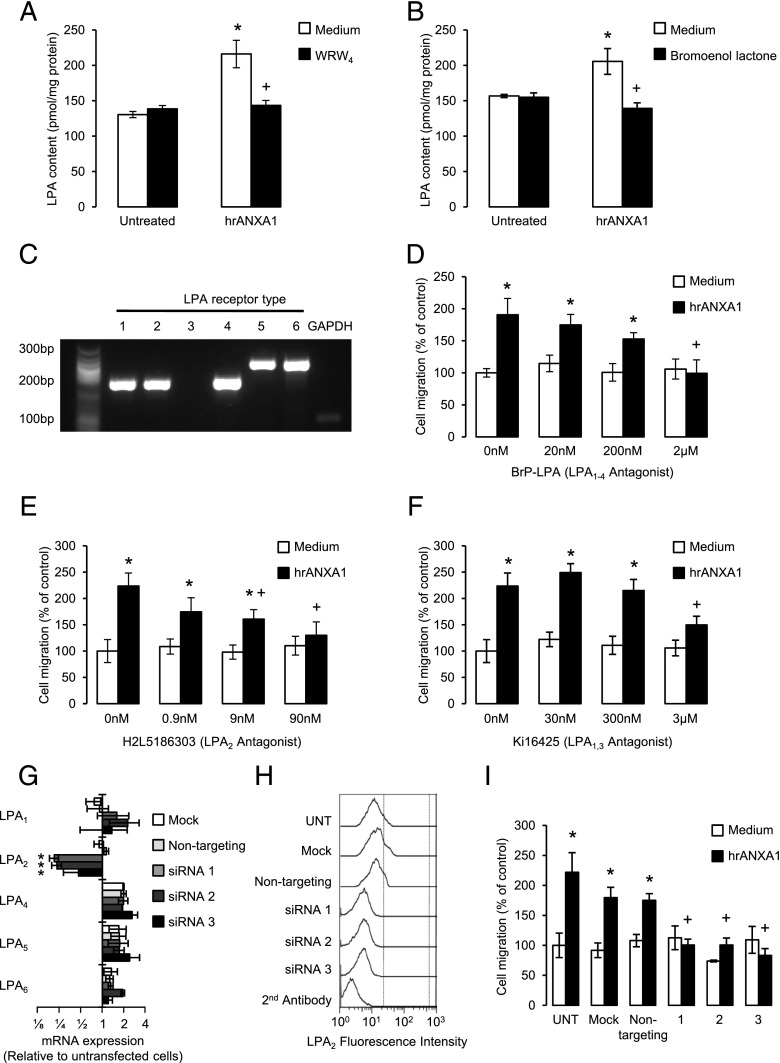
Chemotaxis of monocytes toward hrANXA1 is dependent upon production of LPA and consequent activation of the LPA_2_ receptor. (**A**) Analysis of human monocyte LPA content 30 min poststimulation with 300 pM hrANXA1 with or without pretreatment for 10 min with the selective ALX/FPR2 antagonist WRW_4_ at 10 μM; data are mean ± SEM of three independent donors. *p < 0.05 versus untreated controls, ^+^*p* < 0.05 versus hrANXA1 treatment. (**B**) Analysis of human monocyte LPA content 30 min poststimulation with 300 pM hrANXA1 with or without pretreatment for 10 min with the iPLA_2_ inhibitor bromoenol lactone at 60 nM; data are mean ± SEM of three independent donors. *p < 0.05 versus untreated controls, ^+^*p* < 0.05 versus hrANXA1 treatment. (**C**) RT-PCR analysis of LPA receptor gene expression in human monocytes alongside GAPDH positive control; image is representative of data from three independent donors. (**D**) Chemotaxis of human monocytes toward 300 pM hrANXA1 with or without 10-min preincubation with the pan-specific LPA_1–4_ receptor antagonist 1-bromo-3(S)-hydroxy-4-(palmitoyloxy)butyl phosphonate; data are mean ± SEM of three independent donors. *p < 0.05 versus medium control, ^+^*p* < 0.05 versus hrANXA1 treatment alone. (**E**) Chemotaxis of human monocytes toward 300 pM hrANXA1 with or without 10-min preincubation with the specific LPA_2_ receptor antagonist H2L5186303; data are mean ± SEM of three independent donors. *p < 0.05 versus medium control, ^+^*p* < 0.05 versus hrANXA1 treatment alone. (**F**) Chemotaxis of human monocytes toward 300 pM hrANXA1 with or without 10-min preincubation with the LPA_1_ and LPA_3_ receptor antagonist Ki16425; data are mean ± SEM of three independent donors. *p < 0.05 versus medium control, ^+^*p* < 0.05 versus hrANXA1 treatment alone. (**G**) Expression of LPA_1_, LPA_2_, LPA_4_, LPA_5_, and LPA_6_ mRNA in human monocytes 48 h after mock transfection, or transfection with a nontargeting siRNA control sequence or one of three independent siRNA constructs specifically targeting LPA_2_, measured using the 2^−ΔΔCt^ method and expressed as relative to untransfected cells; data are mean ± SEM of three independent donors. *p < 0.05 versus mock transfected by Kruskal–Wallis analysis. (**H**) Typical flow cytometry profiles of surface LPA_2_ receptor expression on untransfected human monocytes or 48 h after mock transfection, or transfection with a nontargeting siRNA control sequence or one of three independent siRNA constructs specifically targeting LPA_2_. (**I**) Chemotaxis toward 300 pM hrANXA1 of untransfected human monocytes or cells 48 h after mock transfection, or transfection with one of three siRNA constructs specifically targeting LPA_2_ or a nontargeting negative control siRNA; data are mean ± SEM of three independent donors. *p < 0.05 versus medium control, ^+^*p* < 0.05 versus migration to hrANXA1 of nontargeting siRNA-transfected cells.

The principal iPLA_2_ product is the phospholipid LPA. As LPA can promote cancer cell migration during metastasis (28), we investigated whether this lipid could mediate the chemotactic actions of ANXA1. Stimulation of human monocytes with hrANXA1 (300 pM; 15 min) significantly enhanced LPA cellular content, an effect blocked by pretreatment with either the ALX/FPR2 antagonist WRW_4_ (Fig. 4A) or the iPLA_2_ inhibitor bromoenol lactone (Fig. 4B), further confirming engagement of this path in ANXA1 signaling in monocytes.

Six distinct receptors have been identified for LPA, of which we detected mRNA transcripts for five (LPA_1_, LPA_2_, LPA_4_, LPA_5_, and LPA_6_) in primary human monocytes (Fig. 4C). Pretreatment of monocytes for 10 min with either the LPA_1–4_ pan-antagonist α-bromomethylene phosphonate-LPA or the LPA_2_ antagonist H2L518603 significantly impaired migration toward hrANXA1, an effect not seen following similar pretreatment with the dual LPA_1_/LPA_3_ antagonist Ki16425, except at the highest concentration tested where selectivity is lost and antagonism for LPA_2_ emerges (Fig. 4D–F). These data strongly suggest that LPA_2_ might be the principal receptor underlying ANXA1-dependent monocyte chemotaxis. This was functionally confirmed using a gene-targeting approach, selectively downregulating LPA_2_ in primary human monocytes. Cells transfected with any of three different LPA_2_-targeting siRNA constructs exhibited significantly reduced chemotaxis to hrANXA1, an effect seen neither upon transfection of cells from the same donors with a nontargeting negative control siRNA nor in mock-transfected cells (Fig. 4G, 4H). Importantly, targeting LPA_2_ did not affect gene products for the other LPA receptors (Fig. 4G).

### LPA acts via mobilization of the actin cytoskeleton

**FIGURE 5.**
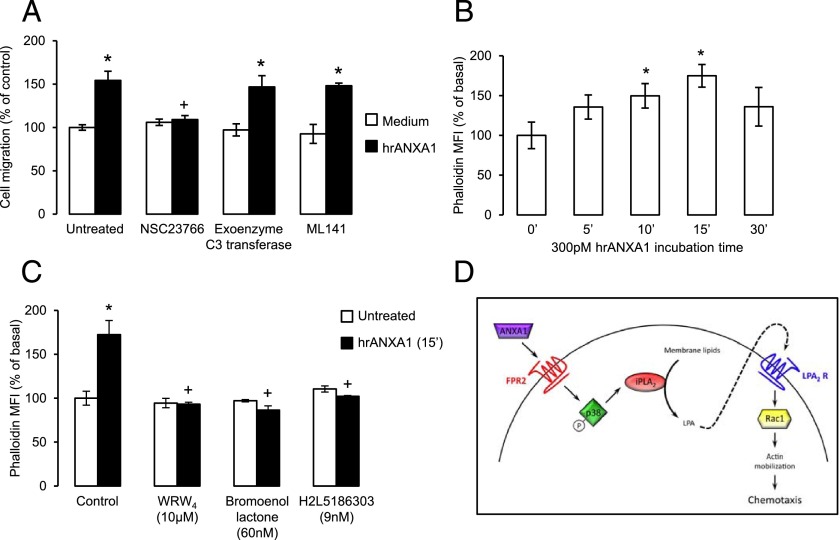
LPA signaling induces actin remodelling via activation of the small G protein Rac1. (**A**) Chemotaxis of human monocytes toward 300 pM hrANXA1 with or without 10-min preincubation with the specific Rac1 inhibitor NSC 23766 (100 μM) or the specific Rho inhibitor exoenzyme C3 transferase (1 μg/ml); data are mean ± SEM of three independent donors. *p < 0.05 versus medium control, ^+^*p* < 0.05 versus hrANXA1 treatment alone. (**B**) Mean fluorescence intensity of phalloidin-AF488–stained human monocytes treated with 300 pM hrANXA1 for 0–30 min; data are mean ± SEM of three independent donors. *p < 0.05 versus untreated control cells. (**C**) Mean fluorescence intensity of phalloidin-AF488–stained human monocytes treated with 300 pM hrANXA1 for 15 min with or without preincubation for 10 min with the ALX/FPR2 antagonist WRW_4_ (10 μM), the iPLA_2_ inhibitor bromoenol lactone (60 nM), or the LPA_2_ antagonist H2L5186303 (9 nM); data are mean ± SEM of three independent donors. *p < 0.05 versus untreated control cells, ^+^*p* < 0.05 versus cells treated with hrANXA1 alone. (**D**) Schematic representation of proposed mechanism by which ANXA1 induces monocyte chemotaxis.

LPA receptors couple to a variety of G proteins leading to activation of the small GTPases Rac1 and Rho (29), well known as important in cytoskeletal rearrangement, a prerequisite for cell migration. As we have shown, ANXA1 treatment can modulate small GTPase activity, including the RhoA–ROCK pathway, in target cells (30), we reasoned that inhibiting these pathways might affect ANXA1-dependent chemotaxis. Pretreatment of monocytes with the Rac1 inhibitor NSC23766 blocked the chemotactic effect of hrANXA1, an effect not observed following pretreatment with either the RhoA inhibitor exoenzyme C3 or the Cdc42 inhibitor ML-141 (Fig. 5A).

To verify that cytoskeletal rearrangement follows exposure to ANXA1, we assessed the degree of actin polymerization in primary human monocytes upon treatment with 300 pM hrANXA1, identifying a clear accumulation of F-actin, most prominent 15 min poststimulation (Fig. 5B). Of relevance, to confirm specificity and integrity of the pathway, F-actin accumulation elicited by hrANXA1 was prevented by monocyte pretreatment with the ALX/FPR2 antagonist WRW_4_, the iPLA_2_ inhibitor bromoenol lactone, or the LPA_2_ antagonist H2L518603 (Fig. 5C).

Together, these data allow us to propose the schematic pathway depicted in Fig. 5D, whereby ANXA1 activation of ALX/FPR2 in CD14^high^CD16^−ve^ monocytes leads, through p38/iPLA_2_ signaling, to LPA formation and downstream activation of the LPA_2_ receptor, in an autocrine or more likely paracrine fashion, ultimately causing actin reorganization and cell chemotaxis.

### ANXA1 acts as a murine monocyte chemoattractant via alx/fpr2/3 in vivo

**FIGURE 6.**
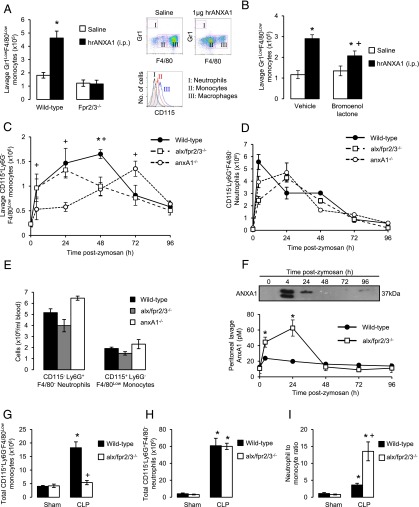
ANXA1 recruits monocytes during inflammation in vivo through the mediation of iPLA_2_. (**A**) Peritoneal lavage monocytes 24 h after i.p. administration of 1 μg hrANXA1 or 100 μl saline to wild-type or alx/fpr2/3^−/−^ mice. *Inset*, Typical flow cytometry profiles of 10,000 events from peritoneal lavages of wild-type mice treated with saline or hrANXA1; gated populations are I, neutrophils; II, monocytes; and III, macrophages. Data are mean ± SEM, *n* = 6. *p < 0.05 versus saline control. (**B**) Peritoneal lavage monocytes 24 h after i.p. administration of 1 μg hrANXA1 or 100 μl saline to wild-type mice with or without pretreatment with 6 mg/kg bromoenol lactone (2 injections, 24 h apart) or vehicle; data are mean ± SEM, *n* = 6. *p < 0.05 versus saline control, ^+^*p* < 0.05 versus vehicle. (**C**) Peritoneal lavage monocytes 0, 4, 24, 48, 72, and 96 h after i.p. administration of 0.5 mg zymosan to wild-type (●), alx/fpr2/3^−/−^ (☐), or AnxA1^−/−^ (○) mice; data are mean ± SEM, *n* = 4. *p < 0.05 for wild-type versus alx/fpr2/3^−/−^, ^+^*p* < 0.05 for wild-type versus AnxA1^−/−^. (**D**) Peritoneal lavage neutrophils 0, 4, 24, 48, 72, and 96 h after i.p. administration of 0.5 mg zymosan to wild-type (●), alx/fpr2/3^−/−^ (☐), or AnxA1^−/−^ (○) mice; data are mean ± SEM, *n* = 4. (**E**) Blood neutrophils and monocytes 48 h after i.p. administration of 0.5 mg zymosan to wild-type alx/fpr2/3^−/−^ or AnxA1^−/−^ mice; data are mean ± SEM, *n* = 4. (**F**) Peritoneal lavage AnxA1 content 0, 4, 24, 48, 72, and 96 h after i.p. administration of 0.5 mg zymosan to wild-type (●) and alx/fpr2/3^−/−^ (☐) mice. *Inset*, Typical Western blot of lavage AnxA1 content 0, 4, 24, 48, 72, and 96 h after i.p. administration of 0.5 mg zymosan to wild-type mice. Data are mean ± SEM, *n* = 4. *p < 0.05 versus wild type. (**G**) Peritoneal lavage monocytes 24 h after cecal ligation and puncture of wild-type and alx/fpr2/3^−/−^ mice; data are mean ± SEM, *n* = 7–8. *p < 0.05 versus sham control, ^+^*p* < 0.05 versus wild type. (**H**) Peritoneal lavage neutrophils 24 h after cecal ligation and puncture of wild-type and alx/fpr2/3^−/−^ mice; data are mean ± SEM, *n* = 7–8. *p < 0.05 versus sham control. (**I**) Peritoneal lavage neutrophil to monocyte ratio 24 h after cecal ligation and puncture of wild-type and alx/fpr2/3^−/−^ mice; data are mean ± SEM, *n* = 7–8. *p < 0.05 versus sham control, ^+^*p* < 0.05 versus wild type.

Having identified ANXA1 as an effective human monocyte chemoattractant in vitro, we investigated whether this novel bioaction would hold true in animal models in vivo. Direct administration of hrANXA1 to the mouse peritoneum (1 μg, i.p. in 100 μl saline) induced a clear recruitment of monocytes to the cavity within 24 h, a response markedly absent in mice lacking the murine equivalents of ALX/FPR2, termed alx/fpr2/3 (Fig. 6A).

Measurement of GFP expression, which in this colony is under the control of the fpr2/3 locus (17), revealed greater gene activity in Ly6C^high^ monocytes than in Ly6C^low^ cells (GFP median fluorescence intensity: Ly6C^high^F4/80^+^, 17.2 ± 1.6 versus Ly6C^low^F4/80^+^, 3.3 ± 0.2, *p* < 0.01 by paired *t* test), indicating that, as with humans, murine classical monocytes are the principal responders to ANXA1. To investigate whether the same pathway elucidated with human cells was operative in the mouse, the effects of the selective iPLA_2_ inhibitor bromoenol lactone were tested. At the dose of 6 mg/kg (−24 h and time 0 i.v.) (31), bromoenol lactone significantly reduced ANXA1-induced monocyte recruitment in wild-type mice, without altering native peritoneal cells (Fig. 6B).

We next investigated whether endogenous ANXA1 played a similar role during more complex inflammatory settings, using zymosan-induced peritonitis as a typical resolving inflammatory response (32). Administration of a mild dose of zymosan (0.5 mg) induced a time-dependent recruitment of monocytes in wild-type mice, peaking between 24- and 48-h administration (Fig. 6C), as reported previously (33). In contrast, both anxA1^−/−^ and alx/fpr2/3^−/−^ mice showed markedly attenuated monocyte recruitment (Fig. 6C), despite high neutrophil extravasation (Fig. 6D), and the presence of similar circulating monocyte numbers as wild-type mice (Fig. 6E). Moreover, comparison of inflammatory exudate cytokines (Table III) revealed not only that anxA1^−/−^ and alx/fpr2/3^−/−^ mice exhibited exaggerated inflammatory reactions compared with wild-type animals, indicated by levels of TNF-α, IL-1β, and IL-10 at the 4-h time point, but that, despite the diminished recruitment of monocytes in the knockout strains, levels of the chemoattractants CCL2, CXCL1, and CCL5 were in fact higher (at 4 h postzymosan) than in wild-type animals. Additionally, to verify whether the reduced monocyte recruitment in alx/fpr2/3^−/−^ mice was due to a failure in production of ANXA1, we measured levels of the protein in peritoneal lavages of wild-type and alx/fpr2/3^−/−^ mice. Both genotypes exhibited an increase in endogenous lavage AnxA1 4 h postzymosan, but this increase was markedly greater in alx/fpr2/3^−/−^ mice and was maintained for at least 24 h (Fig. 6F).

**Table III.** Peritoneal lavage chemokines and cytokines after zymosan-induced peritonitis in wild-type, Fpr2/3^−/−^, and AnxA1^−/−^ mice

		0 h	4 h	24 h	48 h	72 h	96 h
MCP-1 (pg/ml)	Wild type	95.9 ± 9.0	9,009.2 ± 933.6	181.6 ± 21.8	121.3 ± 7.8	104.3 ± 3.4	137.6 ± 18.6
	Fpr2/3^−/−^	112.6 ± 7.4	11,534.9 ± 493.4*	155.9 ± 4.6	85.9 ± 3.3	99.2 ± 3.6	90.8 ± 7.9
	AnxA1^−/−^	106.4 ± 8.8	9,317.6 ± 404.9^+^	160.1 ± 20.7	104.7 ± 12.3	131.3 ± 28.1	108.5 ± 9.4
KC (CXCL1)	Wild type	31.5 ± 6.1	3,441.8 ± 1,047.8	29.6 ± 2.8	29.5 ± 2.7	26.0 ± 3.3	27.0 ± 1.6
	Fpr2/3^−/−^	10.6 ± 0.6	15,259.4 ± 4,831.7*	17.1 ± 4.8	12.3 ± 2.5	10.0 ± 0.5	15.0 ± 3.3
	AnxA1^−/−^	26.9 ± 3.9	4,591.2 ± 2,147.6^+^	29.0 ± 7.7	25.0 ± 2.3	21.3 ± 1.1	20.4 ± 2.2
RANTES (CCL5)	Wild type	4.3 ± 0.6	115.3 ± 28.9	7.2 ± 1.1	8.4 ± 1.8	3.5 ± 1.0	5.1 ± 0.8
	Fpr2/3^−/−^	4.1 ± 1.2	105.4 ± 32.4	4.1 ± 0.4	6.3 ± 1.2	2.2 ± 0.4	4.1 ± 0.2
	AnxA1^−/−^	4.6 ± 0.4	92.0 ± 17.7	5.5 ± 0.5	14.2 ± 5.4	4.0 ± 0.7	4.2 ± 0.5
TNF-α (pg/ml)	Wild type	n.d.	15.2 ± 3.4	n.d.	n.d.	n.d.	n.d.
	Fpr2/3^−/−^	n.d.	90.7 ± 12.9*	n.d.	n.d.	n.d.	n.d.
	AnxA1^−/−^	n.d.	40.4 ± 6.7*	n.d.	n.d.	n.d.	n.d.
IL-1β (pg/ml)	Wild type	n.d.	86.0 ± 10.7	13.3 ± 1.8	9.7 ± 2.1	n.d.	11.7 ± 3.8
	Fpr2/3^−/−^	17.7 ± 8.0	249.7 ± 54.6*	n.d.	n.d.	n.d.	13.4 ± 4.1
	AnxA1^−/−^	13.6 ± 4.9	109.3 ± 19.6^+^	18.4 ± 7.0	13.3 ± 6.2	n.d.	n.d.
IL-10 (pg/ml)	Wild type	214.6 ± 12.8	1,126.7 ± 185.9	155.5 ± 31.3	81.7 ± 21.8	66.7 ± 19.9	82.2 ± 6.0
	Fpr2/3^−/−^	187.9 ± 49.6	468.7 ± 123.8*	127.9 ± 43.2	122.0 ± 27.7	48.6 ± 3.1	62.4 ± 13.8
	AnxA1^−/−^	191.5 ± 34.6	486.5 ± 190.4*	61.5 ± 14.2	66.7 ± 21.3	51.4 ± 27.9	72.5 ± 39.6

Data are mean ± SEM. *n* = 4.

* *p* < 0.05 versus wild-type, ^+^*p* < 0.05 versus Fpr2/3^−/−^.

n.d., below detection limit.

Confirmation of the importance of this pathway in inflammation was provided by use of the cecal ligation and puncture model of sepsis (19). Wild-type animals exhibited significant recruitment of monocytes 24 h postsurgery, but this was markedly absent in alx/fpr2/3^−/−^ mice, despite similar neutrophil recruitment in both genotypes (Fig. 6G, 6H). Experiments were terminated for ethical reasons at 24 h postsurgery, at which point the neutrophil to monocyte ratio (a hallmark of resolution) was significantly greater in septic alx/fpr2/3^−/−^ mice than their wild-type counterparts (Fig. 6I), further indicating a failure of inflammatory resolution.

These experiments indicate that ALX/FPR2 activation by endogenous ligands or exogenous ANXA1 can promote monocyte recruitment, an effect that, at least following pharmacological administration, relies on the mouse orthologs of ALX/FPR2 and iPLA_2_ activation. Characterization of the response in alx/fpr2/3^−/−^ mice suggests the existence of an altered loop, as evidenced by abnormal levels of AnxA1—and specific chemokines—in inflammatory exudates.

### Apoptotic neutrophils are the principal source of ANXA1 in inflammation

**FIGURE 7.**
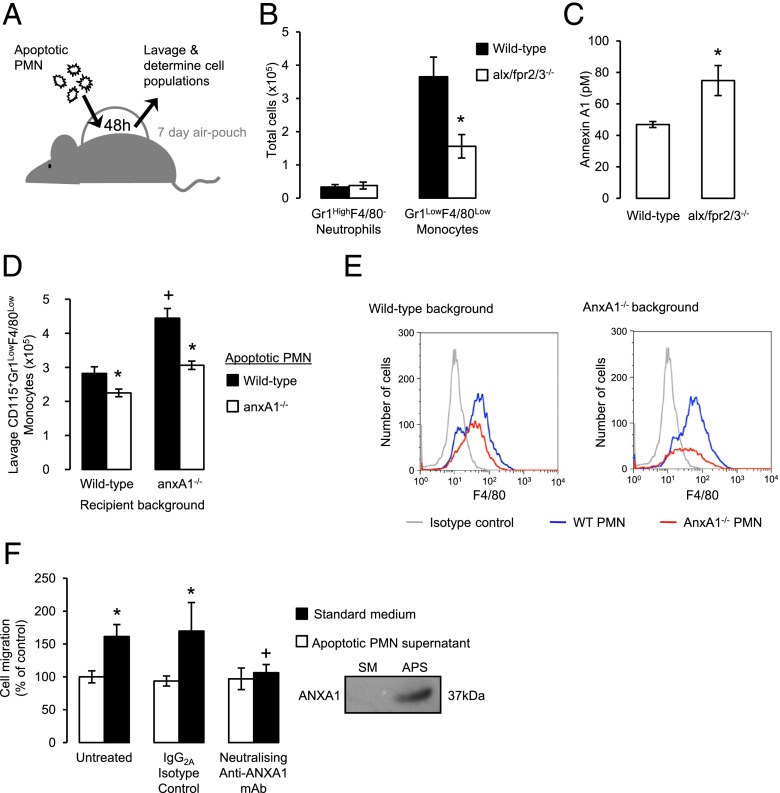
Apoptotic neutrophils are the principal source of AnxA1-inducing monocyte recruitment in vivo. (**A**) Schematic representation of design of air-pouch experiments. (**B**) Air-pouch lavage neutrophils and monocytes 48 h after the administration of 10^6^ murine polymorphonuclear cells previously rendered apoptotic through overnight incubation with 1 μg/ml actinomycin D to wild-type or alx/fpr2/3^−/−^ mice; data are mean ± SEM, *n* = 6. *p < 0.05 versus wild type. (**C**) Air-pouch lavage AnxA1 24 h after the administration of 10^6^ apoptotic murine polymorphonuclear cells to wild-type or alx/fpr2/3^−/−^ mice; data are mean ± SEM, *n* = 6. *p < 0.05 versus wild type. (**D**) Total lavage monocytes 24 h after administration of 10^6^ apoptotic murine polymorphonuclear cells from wild-type or AnxA1^−/−^ mice to air pouches borne by wild-type or AnxA1^−/−^ mice; data are mean ± SEM, *n* = 6. *p < 0.05 versus animals of the same genotype administered wild-type polymorphonuclear cells, ^+^*p* < 0.05 versus wild-type animals receiving wild-type polymorphonuclear cells. (**E**) Representative flow cytometry histograms showing recruitment of F4/80^+^ murine monocytes. (**F**) Chemotaxis of human monocytes toward cell-free supernatant from apoptotic polymorphonuclear cells, treated with either a neutralizing anti-ANXA1 mAb, or its IgG_2A_ isotype control (50 ng/ml in both cases). *Inset*, Typical Western blot of ANXA1 content in standard medium (SM) or apoptotic polymorphonuclear cell supernatant (APS). Data are mean ± SEM of three independent donors. *p < 0.05 versus medium control, ^+^*p* < 0.05 versus isotype control alone.

Having established that ANXA1 can serve as a monocyte chemoattractant in vitro and in vivo, we investigated the cellular source during ongoing inflammation: neutrophils were our prime suspects, as they are known to exhibit enhanced AnxA1 gene expression upon tissue recruitment (34) and to release the protein upon apoptosis (13). Dorsal air pouches were created on male wild-type and alx/fpr2/3^−/−^ mice, into which apoptotic murine neutrophils were injected (Fig. 7A). After 48 h, significant monocyte recruitment was seen in wild-type animals, but this was impaired (by ∼50%) in alx/fpr2/3^−/−^ mice (Fig. 7B). This reduced response was not due to inadequate AnxA1 production, as pouch lavage AnxA1 levels were even greater in alx/fpr2/3^−/−^ mice than in their wild-type counterparts (Fig. 7C).

To confirm that apoptotic neutrophils were the principal source of ANXA1, we performed crossover experiments in which apoptotic neutrophils from either wild-type or AnxA1^−/−^ mice were injected into dorsal air pouches borne on either AnxA1^−/−^ or wild-type animals. AnxA1^−/−^ mice had significantly greater monocyte recruitment than wild-type animals, suggestive of enhanced inflammatory reactivity to apoptotic cells, as has been reported previously (18). However, more relevant in this study, animals treated with AnxA1^−/−^ apoptotic neutrophils showed significantly lower monocyte recruitment (∼80% reduction) than animals treated with wild-type neutrophils, regardless of their genetic background (Fig. 7D, 7E). As final validation of ANXA1 as the principal chemoattractant from apoptotic neutrophils, we exposed human monocytes to supernatant from apoptotic human polymorphonuclear cells treated with either a neutralizing anti-ANXA1 mAb (35) or its isotype control. Apoptotic polymorphonuclear cell supernatant was a potent monocyte chemoattractant, but this activity was significantly inhibited by inclusion of the anti-ANXA1 Ab, but not the isotype control (Fig. 7F).

Collectively, these findings strongly indicate effete, apoptotic neutrophils as the principal reservoir for ANXA1 in an inflammatory reaction, and are thus important recruiting agents for monocytes to orchestrate the second, resolving phase of acute inflammation.

## Discussion

Efficient and timely monocyte recruitment is a critical step in acute inflammation, enabling the clearance of effete neutrophils and orderly progression toward resolution. In this study, we reveal a novel role for the proresolving protein ANXA1 in this process, identifying its ability to recruit CD14^high^CD16^−^ classical monocytes. Moreover, to our knowledge, we reveal for the first time a functional circuit centered on this protein, signaling via ALX/FPR2 to activate production of LPA through iPLA_2_. Although LPA has been shown to have a role in cancer cell metastasis (28), recent evidence indicates its importance in the monocyte/macrophage response to CCL2 (27, 36), and, together with the present work, this identifies an important role for the endogenous lipid in physiological inflammatory resolution.

A causal link between tissue-infiltrated neutrophils and consequent monocyte recruitment has long been established, with neutrophil-derived soluble factors being important monocyte attractors (7). Although several such candidate chemoattractants are known, these are principally proinflammatory and released by viable, activated neutrophils (7). ANXA1 comprises ∼2–4% of total neutrophil protein (12) and, significantly, is released to a substantial degree upon apoptosis (13). Furthermore, migrated neutrophils upregulate AnxA1 gene activity (34), contributing to the abundant presence of this protein in exudates, as shown in rodent (37) and human (22) settings. In all cases, we propose AnxA1 as optimally placed to signal the presence of effete neutrophils and the need for their clearance—a critical step in resolution. Our data confirm the central role for the ANXA1–ALX/FPR2 pathway in resolution, and add modulation of monocyte recruitment to its described proresolving abilities, that is, limitation of neutrophil extravasation, induction of neutrophil apoptosis, and promotion of apoptotic cell phagocytosis (24).

The comparison of ANXA1 and LL-37 as monocyte chemoattractants is intriguing, given that both signal via ALX/FPR2 to recruit CD14^high^CD16^−^ monocytes. However, whereas LL-37 has clearly been shown to exert a proinflammatory influence upon monocytes, for example, inducing release of IL-1β (38) and IL-8 (39), we and others have shown ANXA1 to induce anti-inflammatory/proresolution effects in these cells, including suppression of IL-6 and TNF-α (40), production of IL-10 (15), and promotion of efferocytosis (13, 35). Notwithstanding this, we propose that future studies will be needed to describe the phenotype of ANXA1-recruited monocytes and hence understand the complex roles played by ALX/FPR2 in regulating monocyte behavior, ultimately leading to a proresolving function of recruited cells. Moreover, this dichotomy in the actions of LL-37 and ANXA1 emphasizes the complex role of ALX/FPR2 in inflammation, which may at least partly be due to its ability to signal either directly or in cooperation with the related classical proinflammatory receptor FPR1 (15). In contrast, it should also be noted that the proinflammatory properties of LL-37 can be overestimated and the final outcome in complex settings is not always predictable; for instance, FPR2-mediated neutrophil migration stimulated by LL-37 is protective in a model of neointimal hyperplasia (41). Similarly, recent elegant studies have shown that exogenous application of the N-terminal fragment of ANXA1, peptide Ac2-26, can prevent chemokine-driven monocyte recruitment to macrovascular atherosclerotic lesions (42); as the short fragment [but not the entire protein (43)] can activate both FPR1 and FPR2, these results emphasize in their entirety the complexity of the interplay between FPR1 and FPR2 in the inflammatory response (15).

Our data strongly support a role for neutrophil-derived ANXA1 in monocyte recruitment, but this protein is not exclusive to neutrophils; it is expressed abundantly by monocytes themselves, as well as epithelial and other cells. Nonetheless, the relative pre-eminence of neutrophil-derived ANXA1 is indicated by our experiments investigating monocyte recruitment to s.c. dorsal air pouches. Administration of apoptotic wild-type neutrophils to AnxA1^−/−^ mice induced monocyte accumulation even greater than that seen in the fully wild-type situation, whereas recruitment of monocytes to wild-type animals in the absence of neutrophil-derived ANXA1 was markedly attenuated, emphasizing that neutrophil-derived ANXA1 is indeed a primary player in this process. The increase in monocyte recruitment quantified in AnxA1^−/−^ mice compared with wild-type animals, regardless of the genotype of cells given, confirms the nonredundant role that endogenous ANXA1 plays in tempering the early phase of inflammation, such as blood-borne leukocyte recruitment, seen by us and others (24).

Although our data clearly implicate iPLA_2_ in ANXA1-driven chemotaxis, the lack of involvement of cPLA_2_ is intriguing, given that previous studies show the need for concerted activation of both enzymes for efficient monocyte migration toward CCL2 (27, 44). Our findings thus not only emphasize the complexity of signaling pathways underlying chemotaxis, but also offer the attractive possibility of selectively enhancing monocyte recruitment to resolving rather than proinflammatory stimuli. Although such a goal requires further study, it may provide novel perspectives in the treatment of chronic inflammatory disease.

Our identification of the central role of iPLA_2_ and consequent LPA production in monocyte chemotaxis toward ANXA1 in vitro and in vivo is of particular interest. Whereas LPA is known to promote cancer cell migration—indeed, it receives much attention as a potential antimetastatic target (28)—the role of this lipid in noncancer settings has been poorly investigated. Production of LPA by the extracellular enzyme autotaxin in secondary lymphoid tissue plays a facilitative role in lymphocyte entry, and exogenously applied LPA can exert promigratory effects on leukocytes (45), but, to our knowledge, this is the first report showing a role for endogenous LPA in innate cell migration. Moreover, although autotaxin-derived LPA has recently been shown to mediate generation of allergic asthmatic inflammation (46), our data would argue for a role of the lipid in resolving processes, highlighting the intricacy of inflammatory control. Receptor selectively could be a clue to these apparently contrasting properties.

To date, six G protein–coupled receptors have been described for LPA, with our data identifying LPA_2_ as a critical actor in monocyte migration toward hrANXA1. The synchronized activation of the two different receptors, ALX/FPR2 and LPA_2_, thus underlying monocyte migration toward ongoing inflammation emphasizes the importance of coordinated signaling networks in resolution. Such harmonized receptor activation appears to be a common feature of ALX/FPR2 behavior, whether this occurs sequentially as described in this work, or through direct receptor association, as seen with, for example, FPR1 (15) or the urokinase receptor (47). Such a network of signaling events centered on ALX/FPR2 may therefore aid in explaining the ability of this receptor to regulate a multitude of fundamental host response processes in inflammation. In other words, we propose that ALX/FPR2 is located high in the hierarchy of proresolving receptors.

Cellular migration ultimately depends upon directed modulation of the cytoskeleton and formation of a leading edge and uropod, an action mediated by small G proteins, and in which the balance of RhoA and Rac1 activity is critical (48). The link between ANXA1 and actin mobilization presented in this work in human monocytes reinforces its relationship to the cytoskeleton, a feature we and others have similarly identified in contexts as diverse as, for example, cerebral endothelial tight junction regulation (30), anterior pituitary hormone release (49), and mast cell degranulation (50). Moreover, whereas LPA_1_ activation has been linked to Rac1-dependent cell motility (51), to our knowledge, this is the first report connecting Rac1 and LPA_2_, adding further detail to our understanding of the role of these receptors in cytoskeletal dynamics.

In conclusion, our data highlight a novel pathway in the self-regulating nature of acute inflammation, with a protein released at high levels from apoptotic neutrophils serving to recruit monocytes that will then remove the same apoptotic cells, protecting healthy surrounding tissues. This is yet more evidence for the importance of ALX/FPR2 in inflammatory regulation, controlling and coordinating the interactions of the various cellular players in this response. It is plausible that these multiple, nonredundant functions of ALX/FPR2 endow this proresolving G protein–coupled receptor with a promising therapeutic potential, as recently discussed (52). In the same vein, our identification of LPA production in ANXA1-driven chemotaxis reveals a novel role for this lipid in resolution, opening up new aspects to its biology and presenting potential pharmacological opportunities for chronic inflammatory conditions.

## Supplementary Material

Data Supplement
