# Supplementary material for: Definition of a Novel Pathway Centered on Lysophosphatidic Acid To Recruit Monocytes during the Resolution Phase of Tissue Inflammation
Source: J Immunol. 2015 Jun 22;195(3):1139–51. doi: 10.4049/jimmunol.1500733 (PMC4505961; doi:10.4049/jimmunol.1500733)
Supplement: Data Supplement [file JI_1500733.zip › JI_1500733_Supplemental_Material_1.pdf]

Supplemental Table I: Chequerboard analysis of neutrophil chemotaxis towards hrANXA1; data are percentage of migrating cells, expressed as mean ± SEM of three independent donors; *p<0.05 *vs.* control migration.

**hrANXA1 in lower well (pM)**	**hrANXA1 in upper well (pM)**			
	0	100	300	1000
0	3.8±0.7	3.1±0.9	3.0±0.8	2.9±0.7
100	2.7±0.7	2.9±0.7	2.9±0.6	3.2±0.6
300	3.4±0.7	2.9±0.6	2.7±0.7	3.5±0.1
1000	2.6±0.4	2.2±0.4	3.3±0.2	3.5±0.5
**fMLP (1μM)**	44.6±1.7			

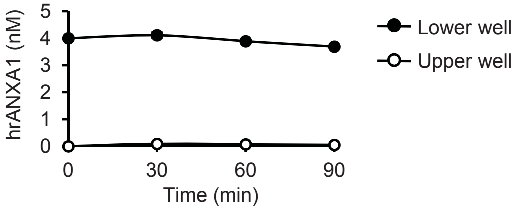

Supplemental figure 1: hrANXA1 gradient stability in chemotaxis assay apparatus. Analysis by specific ELISA of hrANXA1 content in lower and upper wells of a 96-well plate format Boyden chamber chemotaxis assay over time, with t=0 starting values of 4nM hrANXA1 in the lower chamber and ANXA1-free medium in the upper; data are mean ± SEM of 6 wells.

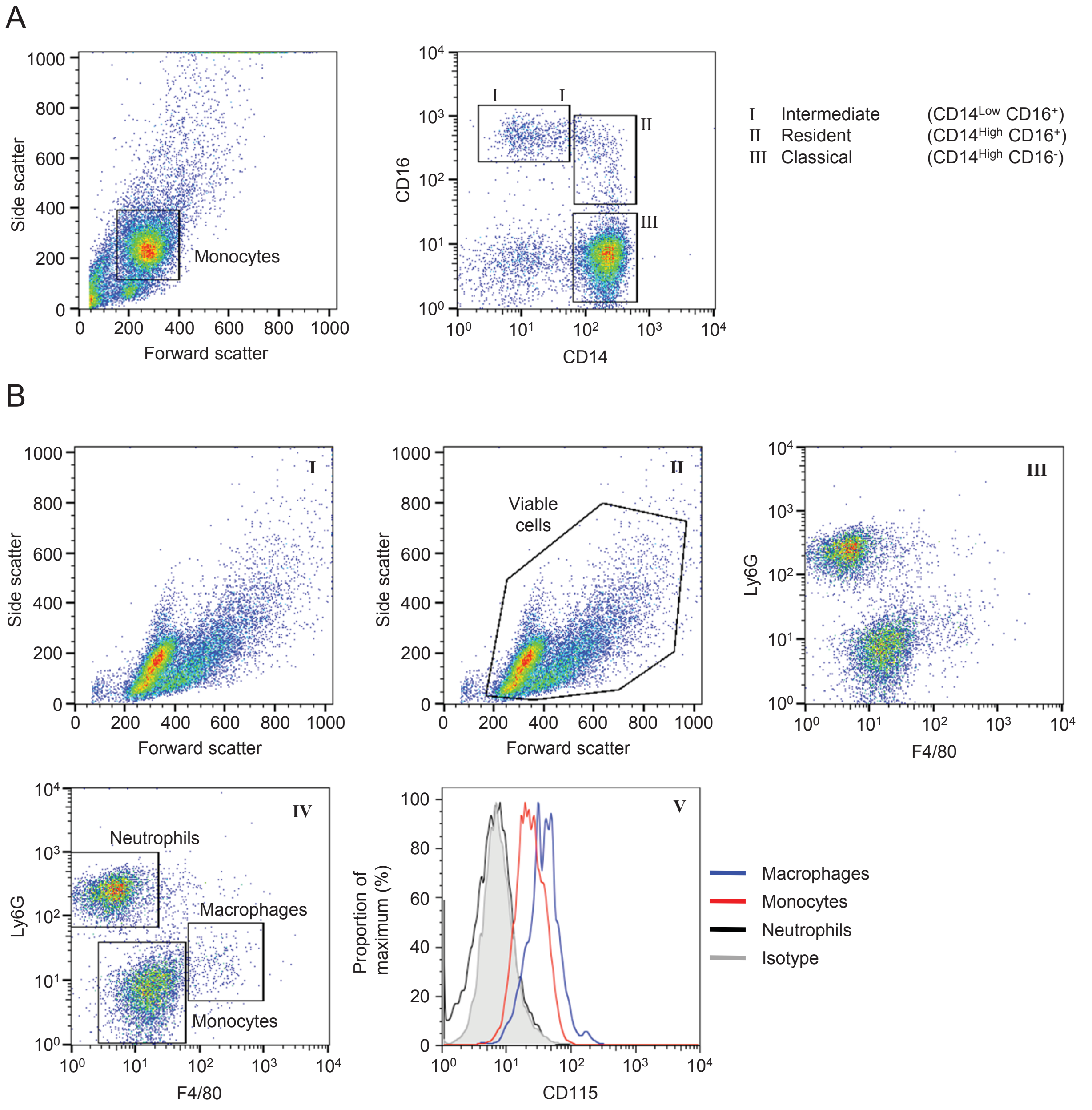

Supplemental figure 2: Flow cytometry gating strategies. A) Gating strategy for the identification of human blood monocyte sub-populations. B) Gating strategy for the identification of murine neutrophils and monocytes in peritonitis, sepsis and air-pouch models.

<u>Supplemental Video Legends</u>

Supplemental video 1: Baseline human primary monocyte motility in a three-dimensional chemotaxis experiment; images taken every 20s for 30min in the absence of chemoattractant, images at 9 fps, scale bar = 10μm

Supplemental video 2: Human primary monocyte motility in a three-dimensional chemotaxis experiment upon exposure to 300pM hrANXA1 (from top of image); images taken every 20s for 30min, images at 9 fps, scale bar = 10μm.
